# Smashing Protein Complex Ions to Bits Reveals Their Structural Organization

**DOI:** 10.1371/journal.pbio.0040287

**Published:** 2006-08-01

**Authors:** Liza Gross

Most cell diagrams ably illustrate the cell's major structural elements, but they can't convey the incredibly dynamic nature of cellular life. One cell can contain millions of ribosomes, each churning out a protein a minute. To make way for new proteins, obsolete proteins must be removed. Short-lived and misshapen proteins get tagged with ubiquitin proteins and sent to the proteasome, a massive enzyme complex that degrades proteins into peptides through a process called proteolysis.

First isolated from yeast, the “26S” proteasome (named after a measure of its macromolecular size) consists of two major subcomplexes, the 20S proteolytic core and the 19S regulatory particle. The 19S particle forms a cap over the 20S core; it has a base that binds to the core and a lid that sits atop the base. After the 19S particle recognizes ubiquitinated substrates and removes their ubiquitin tag, they're unfolded and sent to the core for degradation. To better understand how this important regulatory complex functions, researchers need precise information on its structure—no small task, given that the lid's many subunits presumably undergo dynamic assembly in the cell.

In a new study, Michal Sharon, Carol Robinson, and colleagues present an innovative approach for identifying the structural composition and organization of intact macromolecular complexes. By combining this approach with chemical cross-linking, which provides additional artificial bonds between the 19S components, the researchers were able to determine the lid's structural organization and gain insight into the likely functional interactions between the components.

Sharon et al. first isolated the intact 19S complexes from yeast, then “electrosprayed” the macromolecules, converting them into ions for mass spectral analysis. Mass spectrometry reveals the protein composition of a sample based on its molecular mass. (Ions travel through a “time-of-flight” analyzer, which times the speed of ions as they travel through an electric field; the sorted ions reach a detector that shows the relative abundance of different masses in the sample as peaks across a spectrum.) The resulting mass spectrum confirmed previous reports that the lid consists of nine protein components, and indicated the presence of an intact complex (roughly 376 kiloDaltons) as well as a smaller subcomplex (roughly 185 kiloDaltons).

To further characterize these components, Sharon et al. used tandem mass spectrometry (MS/MS), a technique that involves multiple steps of sorting ions to isolate and analyze the complex's components. The ionized sample accelerates through a gas-filled “collision chamber.” As multiple collisions yield increasing energy, the ionized sample (precursor ion) dissociates into “product ions” (individual subunits). Using this approach on the intact lid, the researchers could infer how the individual pieces of the lid fit together by identifying which of the components stuck together longer. From one round of MS/MS, they identified the two proteins Rpn9 and Rpn12. Then a second, higher energy round of MS/MS triggered the dissociation of a third protein, Rpn6. The researchers concluded that Rpn6, Rpn9, and Rpn12 interact weakly around the lid's periphery. Yet another round of MS/MS, this time on the lid subcomplex, identified Rpn5, Rpn6, and Rpn9 as subunits that can form different interactions with Rpn8, a subunit that had not been detected by itself. Finally, using a technique called chemical cross-linking (which creates covalent bonds between neighboring proteins so they can be examined), the researchers identified additional interaction partners within the lid.

Based on the results of their MS/MS and cross-linking experiments, along with previously published data, the researchers proposed a model of how all these components come together to form the lid complex. The lid consists of two subcomplexes—one comprising Rpn5, Rpn6, Rpn8, and Rpn9; the other of Rpn3, Rpn7, Rpn12, and Sem1—with a link forged between Rpn5 and Rpn3. Rnp11, the enzyme responsible for coupling deubiquitination with degradation, is likely recruited by the first subcomplex. These insights into the configuration and possible role of the lid's subunits will facilitate future investigations into the function and regulation of the 19S lid. And this technique—which can identify the subunit organization of intact complexes based on their mass spectra—should illuminate the architecture of a diverse range of macromolecules still uncharacterized.

## 

**Figure:**
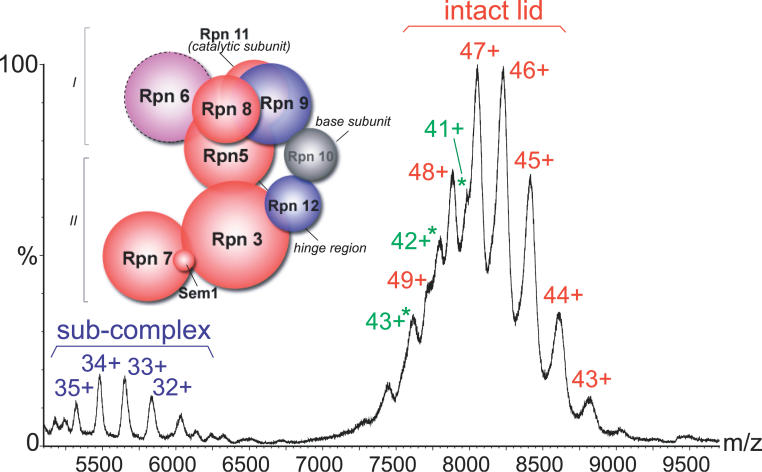
Mass spectrum of the 19S lid complex isolated from yeast cells. The spectrum shows a series of peaks, labeled with charge states in red, corresponding to the nine protein components of the lid.

